# Targeting the PELP1-KDM1 axis as a potential therapeutic strategy for breast cancer

**DOI:** 10.1186/bcr3229

**Published:** 2012-07-19

**Authors:** Valerie Cortez, Monica Mann, Seshidhar Tekmal, Takayoshi Suzuki, Naoki Miyata, Cristian Rodriguez-Aguayo, Gabriel Lopez-Berestein, Anil K Sood, Ratna K Vadlamudi

**Affiliations:** 1 Department of Obstetrics and Gynecology, University of Texas Health Science Center, San Antonio, TX 78229, USA; 2 Department of Cell and Structural Biology, University of Texas Health Science Center, San Antonio, TX 78229, USA; 3 Graduate School of Medical Science, Kyoto Prefectural University of Medicine, 13 Taishogun Nishitakatsukasa-Cho, Kita-ku, Kyoto 403-8334, Japan; 4 PRESTO, Japan Science and Technology Agency, 4-1-8 Honcho Kawaguchi, Saitama 332-0012, Japan; 5 Graduate School of Pharmaceutical Sciences, Nagoya City University, 3-1 Tanabe-dori, Mizuho-ku, Nagoya, Aichi 467-8673, Japan; 6 Department of Gynecologic Oncology, University of Texas MD Anderson Cancer Center, Houston, TX 78030, USA; 7 Center for RNA Interference and Non-coding RNA, University of Texas MD Anderson Cancer Center, Houston, TX 78030, USA; 8 Department of Cancer Biology, University of Texas MD Anderson Cancer Center, Houston, TX 78030, USA; 9 Cancer Therapy and Research Center, University of Texas Health Science Center, San Antonio, TX 78229, USA

## Abstract

**Introduction:**

The estrogen receptor (ER) co-regulator proline glutamic acid and leucine-rich protein 1 (PELP1) is a proto-oncogene that modulates epigenetic changes on ER target gene promoters via interactions with lysine-specific histone demethylase 1 (KDM1). In this study, we assessed the therapeutic potential of targeting the PELP1-KDM1 axis *in vivo *using liposomal (1,2-dioleoyl-*sn*-glycero-3-phosphatidylcholine; DOPC) siRNA to downregulate PELP1 expression and KDM1 inhibitors, pargyline and *N*-((1S)-3-(3-(trans-2-aminocyclopropyl)phenoxy)-1-(benzylcarbamoyl)propyl)benzamide using preclinical models.

**Methods:**

Preclinical xenograft models were used to test the efficacy of drugs *in vivo*. Ki-67 and terminal deoxynucleotidyl transferase dUTP nick end-labeling immunohistochemical analysis of epigenetic markers was performed on tumor tissues. The *in vitro *effect of PELP1-KDM axis blockers was tested using proliferation, reporter gene, chromatin immunoprecipitation and real-time RT-PCR assays. The efficacy of the KDM1 targeting drugs alone or in combination with letrozole and tamoxifen was tested using therapy-resistant model cells.

**Results:**

Treatment of ER-positive xenograft-based breast tumors with PELP1-siRNA-DOPC or pargyline reduced tumor volume by 58.6% and 62%, respectively. In a postmenopausal model, in which tumor growth is stimulated solely by local estrogen synthesis, daily pargyline treatment reduced tumor volume by 78%. Immunohistochemical analysis of excised tumors revealed a combined decrease in cellular proliferation, induction of apoptosis and upregulation of inhibitory epigenetic modifications. Pharmacological inhibition of KDM1 *in vitro *increased inhibitory histone mark dimethylation of histone H3 at lysine 9 (H3K9me2) and decreased histone activation mark acetylation of H3K9 (H3K9Ac) on ER target gene promoters. Combining KDM1 targeting drugs with current endocrine therapies substantially impeded growth and restored sensitivity of therapy-resistant breast cancer cells to treatment.

**Conclusion:**

Our results suggest inhibition of PELP1-KDM1-mediated histone modifications as a potential therapeutic strategy for blocking breast cancer progression and therapy resistance.

## Introduction

Breast cancer accounts for over one-quarter of all cancer diagnoses, with an estimated 200,000 new cases annually [1]. Despite recent advances in diagnosis and treatment strategies, nearly 40,000 women will die of this disease in 2011 [1]. The hormone-dependent nature of breast cancer and the important role of estrogen receptor alpha (ERα) in initiation and progression supported development of pharmacologic agents to either reduce circulating estrogen levels or modulate ERα functions [2,3]. Targeted endocrine therapies significantly reduce mortality in patients with hormone-responsive (ERα-positive) tumors. However, both *de novo *and acquired therapy resistance limits treatment efficacy [4].

ERα transcriptional activity is not only regulated by steroid hormones alone but also requires co-regulatory proteins [5,6]. Following hormone stimulation, multiprotein complexes containing ERα co-regulators and transcriptional regulators assemble to regulate gene transcription [6]. ERα co-regulatory proteins are tightly regulated under normal conditions, with misexpression primarily reported in the literature in association with a number of disease states. Over one-third of the nearly 300 distinct co-regulators identified are overexpressed or underexpressed in human cancers; 38% of co-regulators are overexpressed in breast cancer [7]. These findings suggest that deregulated co-regulator expression may promote carcinogenesis and/or progression of endocrine-related cancers. ERα-associated co-regulator misexpression contributes to ERα activity and often correlates with poor prognosis [8,9]. Consequently, co-regulator expression represents an indirect means of targeting ERα activity.

Estrogen-induced breast carcinogenesis is characterized by aberrant histone modifications [10]. Ligand-bound ERα promotes various histone modifications on target gene promoters and such modifications are facilitated by ERα co-regulatory proteins. Regulatory effects of histone acetylation and phosphorylation have been extensively characterized. However, the role of histone methylation remains understudied. Unlike acetylation, which generally correlates with gene activation, the consequences of histone methylation are site dependent. For example, histone H3 lysine 4 dimethylation (H3K4me2) on ERα target gene promoters correlates with transcriptional activation, while lysine 9 dimethylation (H3K9me2) associates with repression [11,12]. Previous studies show recruitment of lysine-specific histone demethylase 1 (KDM1) to a significant fraction of ERα target genes [13]. Unlike genetic alterations, epigenetic changes are reversible and therefore represent a promising therapeutic target.

Emerging evidence implicates a functional role of ERα co-regulator proline glutamic acid and leucine-rich protein 1 (PELP1) in the oncogenic properties of cancer cells [14]. PELP1 deregulation occurs within several hormone-responsive malignancies including breast cancer, ovarian cancer and prostate cancer [15]. In a subset of human breast tumors, both PELP1 expression and localization are altered [16]; expression during breast cancer progression is associated with more invasive disease [16,17]. In a preclinical study of ERα-positive breast cancer patients, PELP1 expression was identified as an independent prognostic biomarker in assessing clinical outcome; elevated expression associated positively with poor prognosis [17]. Acting as a scaffolding protein, PELP1 coordinates various signaling pathways with ERα by modulating interactions with known oncogenes and cytosolic kinases [15]. PELP1 deregulation correlates with increased aromatase expression resulting in tumor proliferation via local estrogen synthesis [14]. Recent studies indicated that PELP1 interaction with KDM1 plays a key role in PELP1-mediated oncogenic functions [18]. Although such findings suggest a role for the PELP1-KDM1 axis in breast cancer progression, the therapeutic potential of targeting the PELP1-KDM1 axis is unknown.

In the present article we target the PELP1-KDM1 axis using a nanoliposomal formulation of PELP1-siRNA-1,2-dioleoyl-*sn*-glycero-3-phosphatidylcholine (DOPC) administered systemically and KDM1 inhibitors in xenograft-based preclinical breast tumor models. Treatment of ERα-positive tumors with PELP1-siRNA-liposomes or pargyline significantly reduced tumor volume. Further, combining KDM1 targeting drugs with current endocrine therapies substantially impeded growth and restored sensitivity of therapy-resistant breast cancer cells. Our data suggest inhibiting PELP1-KDM1-mediated histone modifications as a potential therapeutic strategy for blocking disease progression and therapy resistance among breast cancer patients.

## Materials and methods

### Cell lines and reagents

Human breast cancer MCF-7 cells were obtained from American Type Culture Collection (Manassas, VA, USA). All of the proposed cells were passaged in the user's laboratory for fewer than 6 months after receipt or resuscitation. MCF-7-PELP1 cells [19], MCF-7-HER2 [20], MCF-7-TamR cells [20] and MCF-7-LTLTca cells [21] have been described earlier. MCF-7 cells transfected with control vector were used as controls. MCF-7-LTLTca and MCF-7-TamR cells were cultured in Phenol red-free RPMI medium containing 5% dextran charcoal-treated serum supplemented with either 1 μmol/l letrozole or 1 μmol/l tamoxifen, respectively.

Estradiol (catalogue number E2257), tamoxifen (catalogue number H7904), androstenedione (catalogue number A9630) and pargyline (catalogue number P8013) were purchased from Sigma (St Louis, MO, USA). *N*-((1S)-3-(3-(trans-2-aminocyclopropyl)phenoxy)-1-(benzylcarbamoyl)propyl)benzamide (NCL-1) was synthesized as previously described [22].

The anti-PELP1 (catalogue number 300-180A) and anti-KDM1 (catalogue number A300-215A) antibodies were purchased from Bethyl Laboratories (Montgomery, TX, USA). Anti-GFP antibody (catalogue number 632381) was purchased from Clontech (Mountain View, CA, USA). Anti-dimethyl-H3K4 (catalogue number 07-030) and anti-H3K9 (catalogue number 07-441) antibodies were purchased from Upstate (Chicago, IL, USA). Anti-acetyl-histone H3 (lys9; catalogue number 9671) was purchased from Cell Signaling (Danvers, MA, USA).

The terminal deoxynucleotidyl transferase dUTP nick end-labeling (TUNEL) kit (catalogue number 11684795910) for apoptosis detection was purchased from Roche (Mannheim, Germany) and Ki-67 anti human Clone MiB-1 antibody (catalogue number M7240) was purchased from Dako (Carpinteria, CA, USA). The PELP1 (catalogue number L004463-00-0050) and KDM1 (catalogue number L-009223-00-0005) nontargeting control (catalogue number D-001810-01-05) SMARTpool siRNA duplexes were purchased from Dharmacon (Lafayette, CO, USA).

### Real-time PCR

Cells were harvested with Trizol Reagent (Invitrogen, Carlsbad, CA, USA) and total RNA was isolated according to the manufacturer's instructions. cDNA synthesis was performed using the Superscript III RT-PCR kit (Invitrogen). Real-time PCR was carried out using a Cepheid SmartCycler II (Sunnyvale, CA, USA) with gene-specific real-time PCR primers. Results were normalized to actin transcript levels and the difference in fold expression was calculated using the ΔΔCT method. The primers for aromatase were 5'-AAATCCAGACTGTTATTGGTGAGAG-3' (sense) and 5'-GTAGCCATCGATTACATCATCTTCT-3' (antisense), and the primers for GREB1C were 5'-GGCAGGACCAGCTTCTGA-3' (sense) and 5'-CTGTTCCCACCACCTTGG-3' (antisense).

### Cell proliferation assay

The cell proliferation rate was measured using a 96-well format with Cell Titer-Glo Luminescent Cell Viability Assay (G7572; Promega). Cells (5×10^3^) were plated in each well of Corning^® ^96-well, clear, flat-bottom, opaque wall microplates and cultured in RPMI media containing 2.5% Dextran Charcoal (DCC) treated serum for 24 hours and followed by treatment with or without estradiol (100 nM/well) for an additional 72 hours. Luminescence was recorded using automatic Fluoroskan Luminometer as per the manufacturer's recommendations. All experiments are carried out using three biological replicates.

### Chromatin immunoprecipitation

The chromatin immunoprecipitation analysis was performed as described previously [18]. Briefly, MCF-7, MCF-7-HER2 and MCF-7-PELP1 cells were cross-linked using 1% formaldehyde, and the chromatin was subjected to immunoprecipitation using the indicated antibodies. Isotype-specific IgG was used as a control. DNA was re-suspended in 50 µl Tris-EDTA buffer (TE) buffer and used for real-time PCR amplification using the gene-specific primers. The primers for aromatase PI.3/11 promoter regions were 5'-CAAGGTCAGAAATGCTGCAA-3' (sense) and 5'-AGCTCCTGTTGCTTCAGGAGG-3' (antisense), and the primers for GREB1C promoter were 5'-TTGTTGTAGCTCTGGGAGCA-3' (sense) and 5'-CAACCAGCCAAGAGGCTAAG-3' (antisense).

### Preparation of liposomal siRNA

For *in vivo *delivery, PELP1 siRNA was incorporated into DOPC nanoliposomes as described previously [23,24]. Briefly, siRNA and DOPC were mixed in excess tertiary butanol at a ratio of 1:10 (w/w) respectively. Subsequently, Tween 20 was added to the mixture at the ratio of 1:19 (Tween 20:siRNA/DOPC). The mixture was vortexed and frozen in an acetone/dry-ice bath and lyophilized. For *in vivo *administration, the mixture was hydrated with 0.9% saline to a concentration of 15 μg/ml, and 200 to 250 μl of the mixture was used for each injection. siRNA for preparation of liposomes were purchased from Sigma. The targeted sequences used were 5'-CCACAGAGCCUGACUCCUA-3' for PELP1 and 5'-UUCUCCGAACGUGUCACGU-3' for control.

### Tumorigenesis assays

All animal experiments were performed after obtaining University of Texas Health Science Center, San Antonio Institutional Animal Care and Use Committee approval, and animals were housed in accordance with the University of Texas Health Science Center, San Antonio institutional protocol for animal experiments.

For tumorigenesis studies, model cells (5×10^6^) were injected into the mammary fatpad of 6-week-old to 7-week-old female nude mice (*n *= 10 per group) as described elsewhere [16]. Athymic nude mice (*nu/nu*) were injected with control MCF-7 cells or with MCF-7 cells that overexpress PELP1 by mixing them with an equal volume of Matrigel^™ ^Matrix (BD Biosciences San Jose, CA, USA). In the premenopausal model, mice received one 60-day release pellet containing 0.72 mg 17β-estradiol (Innovative Research of America, Sarasota, FL, USA) 1 week before implantation of cells. For the postmenopausal model, mice were subjected to ovariectomy 1 week prior to tumor cell inoculation. Owing to the deficiency of adrenal androgens in this model, athymic mice were supplemented with subcutaneous injections of the aromatase substrate androstenedione (100 µg/day) for the duration of the experiment as described for the postmenopausal model [25].

To examine the effects of PELP1 siRNA therapy on tumor growth, treatment was initiated 1 week after intraperitoneal injection of tumor cells. Mice were randomly assigned to two groups (*n *= 10 mice per group): control siRNA-DOPC (150 μg/kg intraperitoneally twice weekly), and PELP1 siRNA-DOPC (150 μg/kg intraperitoneally twice weekly). The mice were monitored daily for adverse toxic effects. Tumor growth was measured with a caliper at weekly intervals, and the volume was calculated using a modified ellipsoidal formula:

$$\text{Tumor}\;\text{volume = 1/2(}L\times W^{2})$$

where *L *is the longitudinal diameter and *W *is the transverse diameter. At the end of each experiment, the mice were euthanized, and the tumors were removed, weighed and processed for immunohistochemistry (IHC) staining.

### Immunohistochemistry

Immunohistochemical analysis was performed as described elsewhere [14]. Briefly, tumor sections were incubated overnight with the primary antibodies PELP1 (1:750), H3K9me2 (1:50), H3K4me2 (1:50), H3K9ac (1:50) and Ki-67 (1:150) in conjunction with proper controls. The sections were then washed three times with 0.05% Tween, incubated with secondary antibody for 1 hour, washed three times with 0.05% Tween in PBS, visualized by 3,3'-diaminobenzidine (DAB) substrateand counterstained with hematoxylin QS (Vector Lab, Burlingame, CA, USA). The proliferative index was calculated as the percentage of Ki-67-positive cells in 10 randomly selected microscopic fields at 40× per slide. TUNEL analysis was performed using the *in situ *Cell Death Detection Kit (Roche, Indianapolis, IN, USA) as per the manufacturer's protocol, and 10 randomly selected microscopic fields in each group were used to calculate the relative ratio of TUNEL-positive cells. The H3K9me2 and H3K4me2 expression of tumors was quantified as 100× the number of positive cells divided by the total number of cells counted under 40× magnification in 10 randomly selected areas in each tumor sample.

### Statistical analysis

Statistical differences among groups were analyzed with either the *t *test or analysis of variance when appropriate using Prism software (Irvine, CA, USA).

## Results

### PELP1 knockdown reduces proliferation and enhances inhibitory epigenetic modifications

We previously demonstrated the feasibility of silencing PELP1 gene expression *in vivo *through systemic administration of PELP1 siRNA [26]. To determine the *in vivo *significance of PELP1 in breast cancer progression, siRNA in a nanoliposomal formulation (PELP1-siRNA-DOPC) was used to silence PELP1 gene expression. Several published studies have validated the delivery and therapeutic efficacy of DOPC-based siRNA nanoliposomes to knock down expression of specific genes *in vivo *[23,27,28]. Adult female athymic *nu/nu *mice received a 17β-estradiol pellet (0.72 mg/pellet, 60-day release) 1 week prior to subcutaneous injection of MCF-7 breast cancer model cells (5×10^6 ^cells) into both flanks. Based on previous dose-response experiments, 150 μg/kg liposomal siRNA every 72 hours effectively downregulates gene expression *in vivo*. Mice bearing xenografts were randomly assigned to receive either control nontargeting siRNA-DOPC or PELP1-siRNA-DOPC via an intraperitoneal route. Following 6 weeks of treatment, mice were euthanized, and tumors were harvested and evaluated for PELP1 expression by IHC.

**Figure 1:**
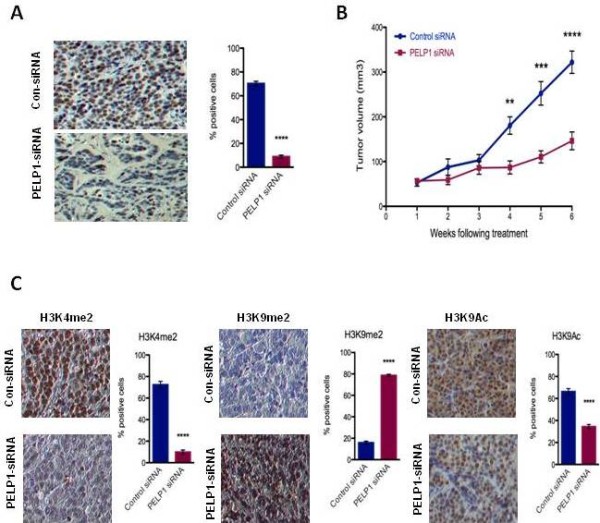
**PELP1 knockdown impedes estrogen-mediated tumor growth**. Nude mice implanted with estrogen pellets were injected subcutaneously with MCF-7 cells. After 4 weeks, xenografts were treated with control-siRNA-DOPC or PELP1-siRNA-DOPC. Tumor growth was measured at weekly intervals. **(A) **Immunohistochemistry (IHC) analysis of PELP1 expression in tumors treated with control or PELP1-siRNA-DOPC (left panel). **(B) **Tumor volume. **(C) **Levels of H3K4me2, H2K9me2 and H3K9ac epigenetic marks were analyzed by IHC in tumors treated with control or PELP1-siRNA-DOPC; quantitation performed as described in Materials and methods. Bar: standard error of the mean. *P < 0.05, **P < 0.001. DOPC, 1,2-dioleoyl-*sn*-glycero-3-phosphatidylcholine; PELP1, proline glutamic acid and leucine-rich protein 1.

Excised tumors from mice treated with PELP1-siRNA-DOPC had markedly low expression of PELP1 (Figure 1A). Compared with controls, mice treated with PELP1-siRNA-DOPC had a significant reduction in tumor volume by 58.6% (*P *< 0.001) (Figure 1B) without causing any observable signs of distress or changes in behavior, mobility or weight loss (data not shown), indicating low treatment toxicity. Tumor growth is regulated by both tumor cell proliferation and apoptosis. We therefore performed immunohistochemical analysis of Ki-67 as a marker of cellular proliferation and TUNEL assay to measure apoptosis. PELP1-siRNA-DOPC treatment significantly decreased tumor cell proliferation and induced apoptosis (Figure S1A,B in Additional file 1) compared with control siRNA-DOPC treatment. Additionally, PELP1-siRNA-DOPC-treated tumors exhibited decreased staining of the activation histone marks H3K4me2 and H3K9ac while increasing the inhibitory epigenetic mark H3K9me2 compared with controls (Figure 1C). Our results indicate that functional PELP1 axis is necessary for optimal proliferation of breast cancer cells and plays a critical role in modulating epigenetic marks on histone tails needed for proliferation *in vivo*.

### Pargyline reduces estrogen-driven proliferation of breast cancer cells

PELP1 is an ERα co-regulator [15] that functions as a proto-oncogene to promote breast tumor cell proliferation [16]. PELP1 deregulation contributes to local estrogen synthesis [14] and PELP1 facilitates ERα crosstalk with HER2 and Src kinases [9]. Recent studies show that PELP1 interaction with lysine-specific demethylase KDM1 plays a key role in PELP1-mediated oncogenic functions via KDM1-mediated epigenetic modifications [18].

**Figure 2:**
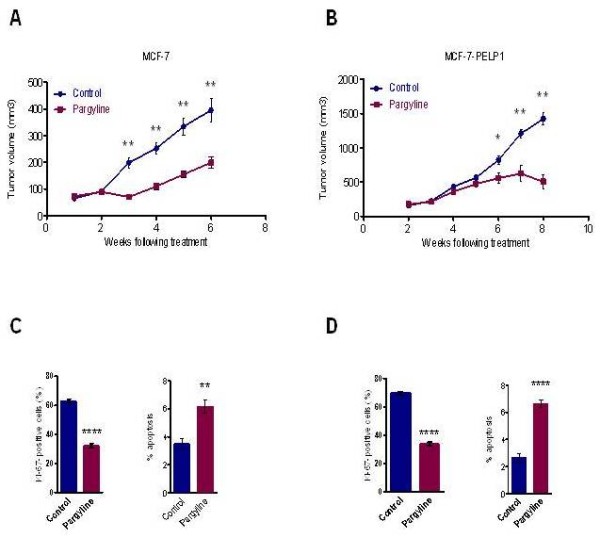
**Pargyline reduces estrogen-mediated tumor growth**. Nude mice implanted with estradiol pellets were injected subcutaneously with (**A**) MCF-7 or (**B**) MCF-7-PELP1 model cells. After 4 weeks, mice were treated with or without pargyline (*n *= 10). Tumor growth was measured at weekly intervals. Tumor volume is shown. Ki-67 expression and TUNEL analysis was done on (**C**) MCF-7 and (**D**) MCF-7-PELP1 xenografts that were treated with or without pargyline. Representative images are shown. Quantitation performed as described in Materials and methods. ****P < 0.0001, **P < 0.001 *P < 0.05. PELP1, proline glutamic acid and leucine-rich protein 1; TUNEL, terminal deoxynucleotidyl transferase dUTP nick end-labeling.

We therefore examined whether the KDM1 blocker pargyline will have clinical utility in blocking estrogen-mediated breast tumorigenesis using preclinical xenograft models. We used pargyline for our *in vivo *studies due to its current US Food and Drug Administraion approval and safety profile [29]. Nude mice (Nu/Nu) were injected with either MCF-7 or MCF-7-PELP1 cells and implanted with estradiol pellet. After establishment of tumors, five mice (10 tumors) per group were treated with pargyline (100 mg/kg in PBS, intraperitoneally, per day) for a period of 6 weeks while control mice received daily PBS injections. MCF-7-PELP1-driven tumors showed aggressive growth compared with MCF-7 tumors (Figure 2A,B). No toxic effects were observed in behavioral changes and body weights were not significantly different between control and pargyline-treated groups (data not shown). Pargyline treatment significantly reduced the tumor volume by 62% and 77% in MCF-7 and MCF-7-PELP1 xenografts, respectively, compared with control groups (Figure 2A,B). Pargyline-treated tumors revealed decreased proliferation, as evidenced by decreased nuclear Ki-67 staining, and exhibited increased apoptosis, as seen by TUNEL positivity (Figure 2C,D). These results suggest that pargyline has therapeutic potential in reducing breast tumor growth.

### Pargyline treatment promotes inhibitory histone methyl marks

KDM1, an enzyme that demethylates both H3K9me2 and H3K4me2, and ER together with PELP1 regulate substrate specificity of KDM1 from H3K4me2 to H3K9me2 [18]. To understand the mechanism, we examined whether paragyline-mediated growth inhibition correlated with the status of the inhibitory H3K9me2 mark at ERα target gene promoters. Pargyline treatment significantly reduced the growth of MCF-7, MCF-7-PELP1 and MCF-7-HER2 cells *in vitro *with a 50% or more reduction in cell proliferation of 4.5 mM, 1.5 mM and 3 mM, respectively, and pargyline (3 mM) inhibited KDM1 activity in an *in vitro *demethylation assay (Figure S2A,B in Additional file 2).

**Figure 3:**
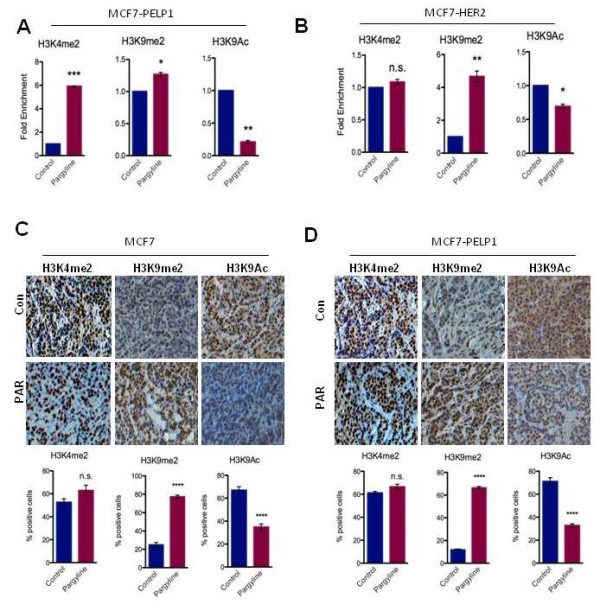
**Pargyline promotes inhibitory epigenetic modifications**. (**A**) MCF-7-PELP1 cells and (**B**) MCF-7-HER2 cells were treated with pargyline (3 mM), and chromatin immunoprecipitation analysis was performed using H3K4me2-specific, H3K9me2-specific or H3K9ac-specific antibodies and the status of epigenetic modifications was analyzed using real-time PCR with the estrogen receptor target gene GREB1C proximal promoter-specific primers (B). Immunohistochemistry analysis of indicated epigenetic marks was done on (**C**) MCF-7 and (**D**) MCF-7-PELP1 xenografts that were treated with or without pargyline. Representative images are shown. Quantitation of staining performed as described in Materials and methods. ****P < 0.0001, ***P < 0.001, **P < 0.01, *P < 0.05. PAR, pargyline; PELP1, proline glutamic acid and leucine-rich protein 1.

MCF-7-PELP1 and MCF-7-HER2 cells were treated with or without pargyline (3 mM) for 72 hours and chromatin was immunoprecipitated using H3K4me2, H3K9me2 and H3K9Ac antibodies. Pargyline treatment increased the H3K4me2 status at the ERα target gene GREB1C promoter in MCF-7-PELP1 cells (Figure 3A). Interestingly, chromatin immunoprecipitation analysis revealed that treatment with pargyline substantially decreased expression of activation mark H3K9ac with a concomitant increase in expression of repressive mark H3K9me2 at GREB1C (Figure 3A). Similarly, KDM1 blockage by pargyline in MCF-7-HER2 model cells increased H3K9me2 expression at the ERα KDM1 target gene GREB1C promoter along with a significantly decreased activation mark H3K9Ac (Figure 3B). Observed inhibitory epigenetic modifications following pargyline inhibitor treatment correlated with decreased estrogen responsive element (ERE) reporter gene activity and relative mRNA expression (Figure S2C,D,E in Additional file 2). IHC analysis of xenograft tumors that were treated with pargyline revealed a substantial increase in H3K9me2 staining (repressive marker) and decreased H3K9Ac (activation marker) staining in pargyline-treated tumors (Figure 3C,D). Collectively, these results indicate that pargyline has the potential to promote inhibitory markers at ERα target genes.

### Pargyline is effective in reducing oncogene-driven tumor growth in a postmenopausal xenograft model

Our earlier studies indicated that the proto-oncogene PELP1 promotes KDM1-driven epigenetic modifications leading to local estrogen synthesis, contributing to cancer cell proliferation and therapy resistance [19]. To examine whether PELP1-driven breast tumors can be therapeutically targeted using pargyline, we performed *in vivo *experiments using a postmenopausal xenograft model. Ovariectomized *nu/nu *mice were injected with MCF-7 or MCF-7-PELP1 cells in an equal volume of Matrigel™ matrix. Athymic mice are deficient in adrenal androgens, and therefore they were supplemented daily with subcutaneous injections of the aromatase substrate androstenedione (100 µg/day) for the duration of the experiment. Under these conditions, injected MCF-7 cells did not form tumors (data not shown). As observed before, MCF-7-PELP1-expressing cells formed tumors in the absence of exogenous estrogen supplementation - suggesting that local derived estrogen supported the growth of MCF-7-PELP1 cells. When the tumor volume reached a palpable stage, mice (*n *= 5, 10 tumors) were either treated with pargyline (100 mg/kg in PBS, intraperitoneally, per day) or vehicle (PBS).

**Figure 4:**
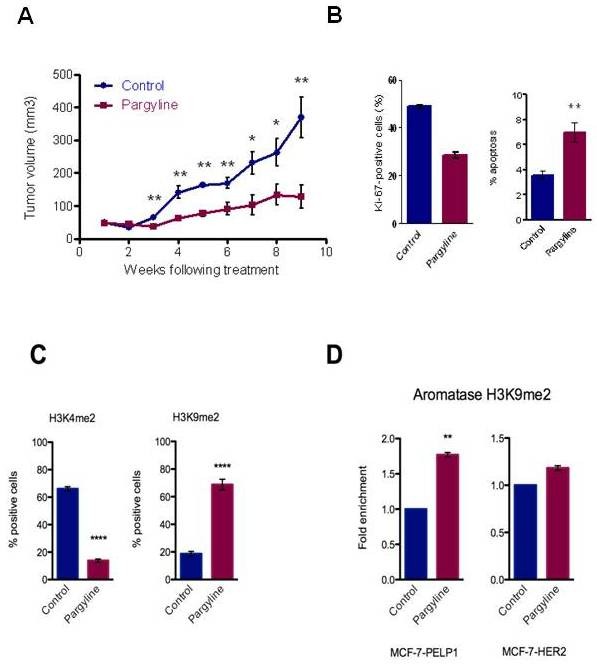
**Pargyline reduces oncogene-driven tumor growth in a postmenopausal xenograft model**. **(A) **Ovarectomized nude mice were injected subcutaneously with MCF-7-PELP1 model cells. After 4 weeks, mice were treated with or without pargyline (*n *= 10). Tumor growth was measured at weekly intervals. Tumor volume is shown. **(B) **Ki-67 expression as a marker of proliferation was analyzed by immunohistochemistry (IHC). Quantitation of Ki-67 staining was performed using the Ki-67 index as described in Materials and methods. **(C) **IHC analysis of indicated epigenetic marks was done on xenograft tumors that were treated with or without pargyline. **(D) **MCF-7-PELP1 and MCF-7-HER2 cells were treated with pargyline (3 mM), and chromatin immunoprecipitation analysis was performed using H3K9me2 antibodies and the status of epigenetic modification was analyzed using real-time PCR with aromatase P1.3/II promoter-specific primers. PELP1, proline glutamic acid and leucine-rich protein 1.

Treatment of PELP1-driven breast tumors with pargyline reduced tumor volume by 78% (Figure 4A). Immunohistochemical analysis of excised xenograft-based tumors revealed a combined decrease in cellular proliferation verified by reduced Ki-67 staining and induced apoptosis as determined by TUNEL positivity (Figure 4B). PELP1-deregulated tumors exhibited excessive H3K4me2 activation markers, and pargyline treatment substantially reduced H3K4me2 staining with a concomitant increase in H3K9me2 inhibitory epigenetic modification (Figure 4C; and Figure S3A in Additional file 3). Earlier studies have shown that both PELP1 and HER2 oncogenes promote activation of ERα target genes as well as local estrogen synthesis via upregulation of aromatase [14,30]. To test whether pargyline treatment promotes inhibitory markers at the aromatase promoter, we have examined the status of the inhibitory H3K9me2 marker after treating both MCF-7-PELP1 and MCF-7-HER2 model cells with pargyline. Results showed a substantial increase in the inhibitory markers at the aromatase PI.3/II promoter (Figure 4D). Accordingly, PELP1-driven xenograft tumors showed increased expression of aromatase and pargyline-mediated growth inhibition correlated with decreased aromatase expression (Figure S3B in Additional file 3). These studies suggest that blockage of KDM1 axis via pargyline has the potential to decrease proto-oncogene PELP1-driven proliferation *in vivo *and pargyline has the potential to reduce growth of oncogene and local estrogen-driven tumors.

### Validation of therapeutic significance of KDM1 using NCL-1

**Figure 5:**
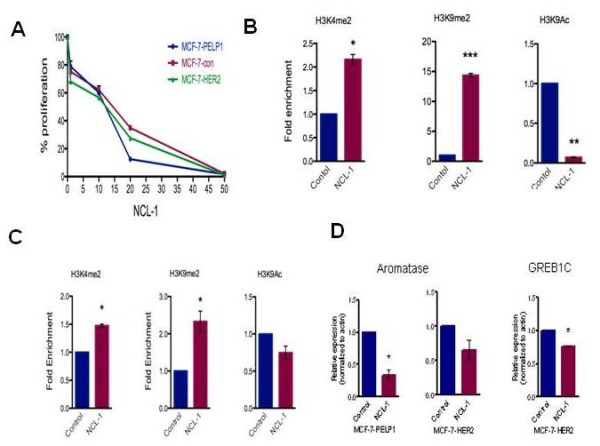
**KDM1 blocker NCL-1 promotes inhibitory epigenetic modifications**. **(A) **Model cells were treated with or without KDM1 inhibitor (NCL-1) and cell proliferation was determined at indicated concentrations using the Cell Titer Glo assay. **(B) **MCF-7-PELP1 cells were treated with or without NCL-1 (10 μM), and chromatin immunoprecipitation (ChIP) analysis was performed using H3K4me2-specific, H3K9me2-specific or H3K9ac-specific antibodies and the status of epigenetic modifications was analyzed using real-time PCR with aromatase P1.3/II promoter-specific primers. **(C) **MCF-7-HER2 cells were treated with or without NCL-1 (10 μM), and ChIP analysis was performed using H3K4me2-specific, H3K9me2-specific or H3K9ac-specific antibodies and the status of epigenetic modifications was analyzed using real-time PCR with estrogen receptor target gene GREB1C proximal promoter-specific primers. **(D) **MCF-7-PELP1 and MCF-7-HER2 cells were treated with NCL-1, total RNA was isolated and expression of aromatase and GREB1 genes was analyzed by quantitative RT-PCR. Error bars indicate ± standard error of the mean. Statistical significance determined by Student's *t *test. ***P < 0.001, **P < 0.01, *P < 0.05. KDM1, lysine-specific histone demethylase 1; NCL-1, *N*-((1S)-3-(3-(trans-2-aminocyclopropyl)phenoxy)-1-(benzylcarbamoyl)propyl)benzamide; PELP1, proline glutamic acid and leucine-rich protein 1.

To independently validate the effect of pharmacological inhibition of KDM1 on the growth of PELP1-driven (MCF-7-PELP1) and HER2-driven (MCF-7-HER2) breast cancer cells, we validated key findings using the recently developed KDM1-specific inhibitor NCL-1 [22]. Both model cells showed a reduction in cell proliferation upon NCL-1 treatment (Figure 5A), with a 50% or more reduction in cell proliferation at a dose range of 12 to 16.5 μM. MCF-7-PELP1 cells were treated with or without NCL-1 for 72 hours and chromatin was immunoprecipitated using H3K4me2, H3K9me2 and H3K9ac antibodies. Chromatin immunoprecipitation analysis using MCF-7-PELP1 cells revealed that treatment with NCL-1 substantially increased H4K4me2, and decreased expression of activation marker H3K9Ac with a concomitant increase in expression of repressive marker H3K9me2 at the aromatase 1.3/II promoter (Figure 5B). Similarly, KDM1 blockage by NCL1 also increased H3K9me2 levels at the ER-KDM1 target gene GREB1C promoter along with a decrease in the levels of activation marker H3K9ac (Figure 5C). Observed inhibitory epigenetic modifications following KDM1 inhibitor treatment correlated with decreased relative mRNA expression, as assessed by quantitative real-time PCR, of aromatase and the ER target GREB1C gene (Figure 5D). Collectively, these results confirm findings we observed following pargyline treatment and suggest that KDM1 blockers have the potential to alter epigenetic changes promoted by oncogenes such as PELP1 and HER2.

### KDM1 blockers reduce proliferation of oncogene-driven and therapy-resistant breast cancer cells

**Figure 6:**
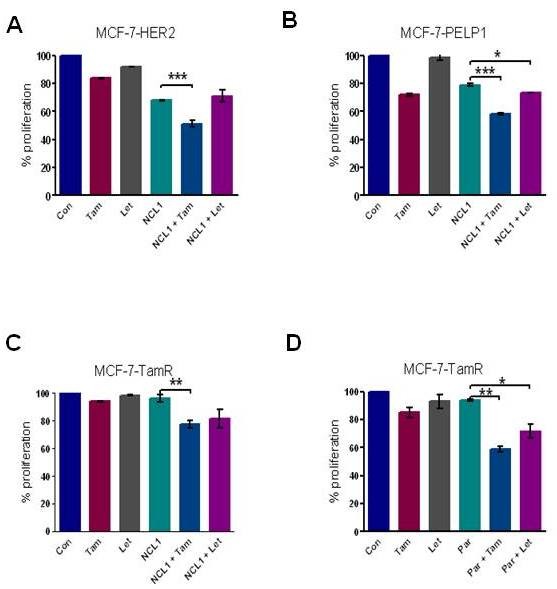
**KDM1 blockers reduce proliferation of oncogene-driven and therapy-resistant breast cancer cells**. Therapy-resistant **(A) **MCF-7-HER2, **(B) **MCF-7-PELP1 and **(C) **MCF-7-TamR model cells were treated with or without NCL-1 (10 μM) alone or in combination with tamoxifen (Tam) or letrozole (Let), and cell proliferation was determined. **(D) **MCF-7-TamR cells were treated with or without KDM1 inhibitor (pargyline, 3 mM) alone or in combination with tamoxifen (Tam) or letrozole (Let), and cell proliferation was determined using the Cell Titer Glo assay. All experimental data points used are generated from three biological replicates. Statistical significance determined by Student's *t *test. ***P < 0.001, **P < 0.01, *P < 0.05. KDM1, lysine-specific histone demethylase 1; NCL-1, *N*-((1S)-3-(3-(trans-2-aminocyclopropyl)phenoxy)-1-(benzylcarbamoyl)propyl)benzamide; PELP1, proline glutamic acid and leucine-rich protein 1.

Deregulation of PELP1 and HER2 contributes to therapy resistance [19]. We therefore examined whether inhibition of the PELP1-KDM1 axis using KDM1 blockers would sensitize therapy-resistant model cells to hormonal therapy. Combinatorial treatment of NCL-1 (10 μM) with tamoxifen (100 nM) significantly reduced the proliferation of three therapy-resistant model cells tested (Figure 6A,B,C). Similarly, combinatorial treatment of pargyline (3 mM) along with tamoxifen showed a significant effect on the growth of therapy-resistant model cells (Figure 6D; and Figure S4 in Additional file 4). Further, siRNA-mediated knockdown of PELP1 or KDM1 expression also substantially reduced the growth of therapy-resistant model cells (Figure S5 in Additional file 5 and Figure S6 in Additional file 6). These results suggested that KDM1 blockers have the potential to reduce growth of oncogene-driven and therapy-resistant breast cancer cells.

## Discussion

Human ERα is implicated in breast cancer initiation and progression. Despite the positive effects of hormonal therapy using anti-estrogens and aromatase inhibitors, *de novo *and/or acquired resistance to endocrine therapies frequently occurs. Alternate therapies are urgently needed to address this major clinical problem. In this study, we tested the hypothesis that deregulation of PELP1 promotes activation of KDM1-driven epigenetic modifications at ERα target genes contributing to cancer proliferation/therapy resistance by testing the therapeutic effect of targeting the PELP1-KDM1 axis. We found that downregulation of PELP1 *in vivo *by PELP1 siRNA liposomes significantly reduced estrogen-mediated breast tumor progression in xenograft model; that drugs targeting KDM1 efficiently reduced PELP1-mediated increases in breast cancer cell proliferation; that KDM1 blockers sensitized therapy-resistant cells to hormonal therapy; that treatment of PELP1 siRNA or KDM1 blockers substantially increased inhibitory histone methyl markers at ER target promoters; and that KDM1 blockers also efficiently reduced breast tumor growth in postmenopausal xenograft models, where tumor growth is driven by local estrogen. Collectively, our results implicate the PELP1-KDM1 axis as a potential therapeutic target for breast cancer.

Changes in ERα co-regulator expression have been demonstrated to substantially contribute to ERα activity and often correlate with poor prognosis. For example, deregulation of the ERα co-regulators SRC3 (AIB1), SRC2 and MTA1 was reported in breast tumors [7]. SRC3 knockout mice studies demonstrated that normal expression of coactivator SRC3 is required for initiation of tumorigenesis by carcinogens and oncogenes [31,32], and overexpression of AIB1 in mouse mammary gland promoted tumorigenesis [33]. Co-regulator PELP1 is shown to function as a proto-oncogene [14] and was recently demonstrated to be an independent prognostic marker for poor breast cancer survival [17]. A recent study suggests that PELP1 mediates androgen receptor activation in the absence of androgens in PCa cells and that disruption of the complex between androgen receptor and PELP1 may be a viable therapeutic strategy in advanced prostate cancer [34]. We found that systemic administration of PELP1 siRNA in a nanoliposomal formulation (PELP1-siRNA-DOPC) significantly reduced breast tumor growth in a xenograft model. IHC analysis of excised xenograft-based tumors revealed a combined decrease in cellular proliferation, induction of apoptosis and upregulation of inhibitory epigenetic modifications of histone H3K9me2 compared with nontreated control groups. These findings suggest that blocking PELP1 expression and/or actions represent an indirect means of targeting the activity of ERα and that blocking the PELP1 axis could have therapeutic implications for reducing breast cancer growth.

Histone methylation plays a vital role in many neoplastic processes and thus represents a valuable therapeutic target [35-37]. Recent evidence suggests activation or repression of estrogen-induced genes depends on the modulation of histone methyl markers on target gene promoters [38]. Histone demethylase KDM1 belongs to a growing number of transcriptional complexes that are implicated in tumorigenesis [39] and is recruited to a significant fraction of ERα target genes [13]. Our previous studies indicate that PELP1 is a novel co-regulator that participates in ERα-mediated chromatin remodeling events via its interactions with KDM1 [18]. Pargyline (Eutonyl, Supirdyl, Eutron) is a US Food and Drug Administration-approved drug to treat depression and vascular hypertension. Several recent studies demonstrated that pargyline has the potential to inhibit KDM1. Here we utilized pargyline to examine whether it has the potential to restore altered epigenetic changes in PELP1-driven breast cancer. Our results showed a significant effect of pargyline in reducing PELP1-driven proliferation. Further, pargyline-treated xenograft tissues showed inhibition of *in vivo *KDM1 activity as can be seen by increased levels of H3K4me2 and H3K9me2, known substrates of KDM1. This proof-of-principle study demonstrated the significance of the PELP1-KDM1 axis in curbing breast cancer progression. However, an extended period of pargyline use at millimolar concentrations may cause side effects. To overcome this possibility, we are currently developing better inhibitors of KDM1 that work efficiently at lower doses with high specificity - we have developed the compound NCL-1, which showed significant activity in the 5 to 10 μM range. Pargyline-mediated inhibition of breast cancer cell growth was independently validated using the more potent KDM1 inhibitor (NCL-1) and also by using PELP1 and KDM1 siRNAs. Recent studies demonstrating the efficacy of KDM1 inhibitors on reducing growth of neuroblastoma [40] and cancer stem cells [41] also corroborate our findings using breast cancer models.

KDM1 can potentially function as a coactivator or co-repressor by demethylating H3K9 or H3K4, respectively, and co-regulators such as PELP1 in conjunction with ERα modulate KDM1 specificity from H3K4me2 to H3K9me2, leading to enhanced ERα target gene activation [42]. As expected, blockage of KDM1 via pargyline or NCL-1 increased both H3K4me2 and H3K9me2 methylation in MCF-7-PELP1 cells. A significant increase in H3K4 methylation in MCF-7-PELP1 model cells also suggests that lack of KDM1 activity facilitated unopposed action of methylases and that PELP1 has the potential to enhance H3K4 methylation via facilitation of H3K4me2 methylases. The latter potential is supported by emerging findings that PELP1 associates with histone methylases [15] and due to the fact that PELP1 knockdown significantly reduced H3K4 methylation (Figure 1C). Pargyline-mediated blockage of KDM1 functions may also strongly increase the repressive H3K9me2 marker, and its subsequent conversion to H3K9me3 marker may lead to reduced recruitment of H3K9 acetyltransferases at specific gene promoters. In support of the second possibility, our ongoing studies identified SETDB1 (an enzyme that facilitates conversion of H3K9me2 to H3K9me3) as a PELP1 interacting protein and showed a reduction of activation marker H3K9Ac in pargyline or NCL-1-treated cells, and earlier studies reported the existence of SETDB1, KDM1 and PELP1 complexes [15]. Blockage of KDM1 functions may provide a favorable environment for SETDB1 to convert H3K9me2 to H3K9me3 under conditions of pargyline treatment. However, future studies are needed to discern these possibilities.

Deregulation of HER2 expression and downstream signaling has emerged as a significant factor in the development of hormonal resistance, and crosstalk with ERα has been shown to promote endocrine therapy resistance [43]. PELP1 interacts with HER2 and is implicated in facilitating ER crosstalk with HER2 signaling pathways [9]. Deregulated PELP1 expression during breast cancer progression is associated with more invasive disease [16,17]. Additionally, PELP1 is shown to contribute to HER2-mediated local estrogen synthesis via increased aromatase expression [14]. Our findings suggest that KDM1 targeting inhibitors (pargyline and NCL-1) are efficient in reducing PELP1-mediated HER2-ERα crosstalk. KDM1 inhibitors efficiently reversed HER2-mediated epigenetic changes and promoted inhibitory histone methyl markers at ERα target genes.

Although mechanisms for hormonal therapy resistance remain elusive, emerging data implicate ERα crosstalk with growth factor pathways and deregulation of co-regulators as major causes of resistance [44]. Earlier studies showed that PELP1 deregulation contributes to therapy resistance [15,45]. Because PELP1 interacts with epigenetic modifier KDM1 [18], in this study we tested whether inhibition of KDM1 by inhibitor pargyline reduced the viability of resistant cells. Combinatorial therapy of anti-estrogen (tamoxifen) with pargyline or NCL-1 showed the most promising therapeutic effect compared with single-agent therapy to inhibit growth of therapy-resistant cells. Results suggest that targeting of the PELP1-KDM1 axis in combination with current endocrine therapies increases therapeutic efficacy and may inhibit or delay development of aromatase inhibitor resistance by promoting favorable epigenetic modifications.

Local estrogen production via deregulated expression of aromatase (Cyp19), the key enzyme in the biosynthesis of estrogen, contributes to tumor progression in postmenopausal women [46]. Aromatase inhibitors are effective in enhancing patient survival although long-term use is limited by systemic side effects and therapy resistance [47]. Recent studies from our laboratory demonstrated PELP1 cooperates with HER2 and modulates epigenetic changes at the aromatase promoter by interacting with lysine-specific demethylase (KDM1), leading to local estrogen synthesis [48]. In this study, we found that KDM1 inhibitors substantially inhibited growth of local estrogen-producing cells (MCF-7-PELP1 and MCF-7-HER2). In the postmenopausal xenograft-based model, treatment with pargyline significantly inhibited the growth of local estrogen-producing PELP1 tumor cells. Our results suggest that drugs targeting the PELP1-KDM1 axis are effective in reversing the methyl modifications at the aromatase promoter that are affected by proto-oncogenes such as PELP1 and HER2 and for blocking growth of local estrogen-producing cells.

## Conclusion

In summary, our data provide the first *in vivo *evidence demonstrating that the PELP1-KDM1 axis is a potential therapeutic target for breast cancer and that targeting the PELP-KDM1 axis has the potential to reduce therapy resistance and local estrogen synthesis. Combining emerging KDM1 targeting drugs with current endocrine therapies, therefore, has the potential to impede growth of co-regulator-deregulated tumors and to restore sensitivity of therapy-resistant breast cancer cells to treatment.

## Abbreviations

DOPC: 1,2-dioleoyl-*sn*-glycero-3-phosphatidylcholine; ER: estrogen receptor; HER2: human epidermal growth factor receptor 2; IHC: immunohistochemistry; KDM1: lysine-specific histone demethylase 1; LTLTca: long-term letrozole treated MCF-7ca; NCL-1: *N*-((1S)-3-(3-(trans-2-aminocyclopropyl)phenoxy)-1-(benzylcarbamoyl)propyl)benzamide; PBS: phosphate-buffered saline; PCR: polymerase chain reaction; PELP1: proline glutamic acid and leucine-rich protein 1; RT: reverse transcriptase; siRNA: small interfering RNA; TamR: tamoxifen-resistant; TUNEL: terminal deoxynucleotidyl transferase dUTP nick end-labeling.

## Competing interests

The authors declare that they have no competing interests.

## Authors' contributions

VC performed the majority of the *in vitro *experiments and xenograft studies, coordinated with all of the team members and drafted the manuscript. MM participated in the KDM1 siRNA studies, demethylase assays and xenograft studies. ST participated in the IHC studies. TS and NM participated in the design and analysis of NCL-1 inhibitor studies. CR-A, GL-B and AKS participated in the design and analysis of siRNA liposome studies. RKV conceived the study, and participated in the design of the project and preparation of final manuscript. All authors read and approved the final manuscript.

## Supplementary Material

Additional file 1 **Figure S1 showing that PELP1 knockdown decreases xenograft tumor proliferation and increases apoptosis**. Nude mice implanted with estrogen pellets were injected subcutaneously with MCF-7 cells. After 4 weeks, xenografts were treated with control-siRNA-DOPC or PELP1-siRNA-DOPC. (A) Ki-67 expression as a marker of proliferation was analyzed by IHC. (B) TUNEL staining was performed as a marker of apoptosis on tumors that were treated with control-siRNA-DOPC or PELP1-siRNA-DOPC. Quantitation was performed as described in Materials and methods. **P < 0.001. Click here for file

Additional file 2 **Figure S2 showing that pargyline reduces proliferation of oncogene-driven breast cancer cells**. (A) Model cells were treated with or without pargyline and cell proliferation was determined at indicated concentrations using the Cell Titer Glo assay. (B) Total histones purified from MCF-7 cells were incubated with purified KDM1 in the presence of the KDM1 inhibitor pargyline (3 mM) or NCL-1 (15 mM) in a standard *in vitro *demethylation assay. Western blot analysis was performed using the total H3 and H3K4-methyl2-specific antibodies. (C) Western analysis of PELP1 levels in MCF-7, MCF-7-PELP1, and MCF-7-HER2 model cells. (D) Model cells were transfected with ERE reporter, after 72 hours, cells were treated with or without pargyline (3 mM) and reporter activity was measured after 12 hours. (E) MCF-7-HER2 cells were treated with pargyline (3 mM), total RNA was isolated and expression of GREB1 genes was analyzed by quantitative RT-PCR. Error bars indicate ± standard error of the mean. Statistical significance determined by Student's *t *test.*P < 0.05. Click here for file

Additional file 3 **Figure S3 showing that pargyline reduces oncogene-driven tumor growth in a postmenopausal xenograft model**. (A) IHC analysis of indicated epigenetic marks was done on xenograft tumors that were treated with or without pargyline. (B) IHC analysis of aromatase expression was done on xenograft tumors that were treated with or without pargyline. ***P < 0.0001. Click here for file

Additional file 4 **Figure S4 showing that pargyline sensitizes therapy-resistant cells to hormonal therapy**. Letrozole-resistant cells (MCF-7-LTLT) were treated with or without KDM1 inhibitor (pargyline) alone or in combination with letrozole (10^-6 ^M) or tamoxifen (10^-7 ^M) and cell proliferation was determined using the Cell Titer Glo assay. **P < 0.001, *P < 0.05. Click here for file

Additional file 5 **Figure S5 showing that PELP1 knockdown reduces the growth of therapy-resistant cells**. Therapy-resistant breast cancer cells were transfected with PELP1 siRNA (50 pmol) or control siRNA (50 pmol), and after 72 hours cell viability was measured by ATP assay (Cell Titer Glo ATP assay; Promega). **P < 0.001. Click here for file

Additional file 6 **Figure S6 showing that KDM1 knockdown reduces the growth of therapy-resistant cells**. (A) Therapy-resistant breast cancer cells were transfected with KDM1 siRNA (50 pmol) or control siRNA (50 pmol), and after 24 hours were treated with tamoxifen (10^-7 ^M). After 72 hours, cell viability was measured by MTT assay. **P < 0.001. (B) Western analysis of KDM1 using total lysates from control-siRNA and KDM1-siRNA transfected cells demonstrating the efficiency of KDM1 knockdown. Click here for file
