# Supplementary figures and images for: Targeting the PELP1-KDM1 axis as a potential therapeutic strategy for breast cancer
Source: Breast Cancer Res. 2012 Jul 19;14(4):R108. doi: 10.1186/bcr3229 (PMC3680946; doi:10.1186/bcr3229)

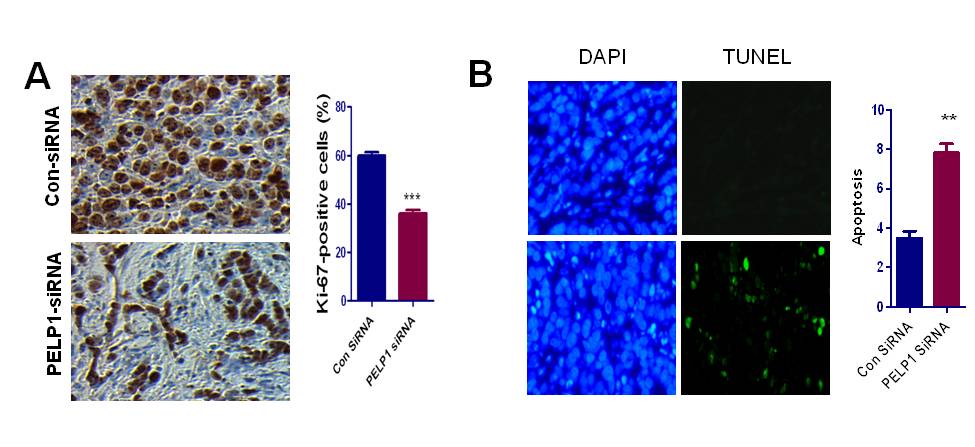

Supplement: Additional file 1 — Figure S1 showing that PELP1 knockdown decreases xenograft tumor proliferation and increases apoptosis. Nude mice implanted with estrogen pellets were injected subcutaneously with MCF-7 cells. After 4 weeks, xenografts were treated with control-siRNA-DOPC or PELP1-siRNA-DOPC. (A) Ki-67 expression as a marker of proliferation was analyzed by IHC. (B) TUNEL staining was performed as a marker of apoptosis on tumors that were treated with control-siRNA-DOPC or PELP1-siRNA-DOPC. Quantitation was performed as described in Materials and methods. **P < 0.001. [file bcr3229-S1.JPEG]

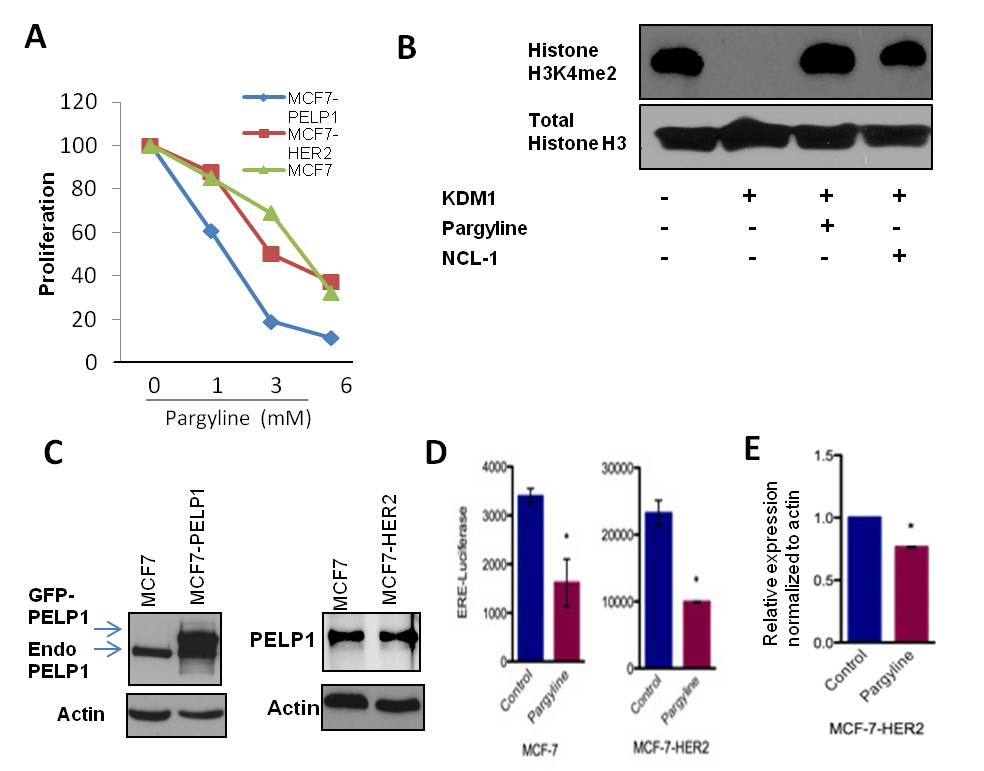

Supplement: Additional file 2 — Figure S2 showing that pargyline reduces proliferation of oncogene-driven breast cancer cells. (A) Model cells were treated with or without pargyline and cell proliferation was determined at indicated concentrations using the Cell Titer Glo assay. (B) Total histones purified from MCF-7 cells were incubated with purified KDM1 in the presence of the KDM1 inhibitor pargyline (3 mM) or NCL-1 (15 mM) in a standard in vitro demethylation assay. Western blot analysis was performed using the total H3 and H3K4-methyl2-specific antibodies. (C) Western analysis of PELP1 levels in MCF-7, MCF-7-PELP1, and MCF-7-HER2 model cells. (D) Model cells were transfected with ERE reporter, after 72 hours, cells were treated with or without pargyline (3 mM) and reporter activity was measured after 12 hours. (E) MCF-7-HER2 cells were treated with pargyline (3 mM), total RNA was isolated and expression of GREB1 genes was analyzed by quantitative RT-PCR. Error bars indicate ± standard error of the mean. Statistical significance determined by Student's t test.*P < 0.05. [file bcr3229-S2.JPEG]

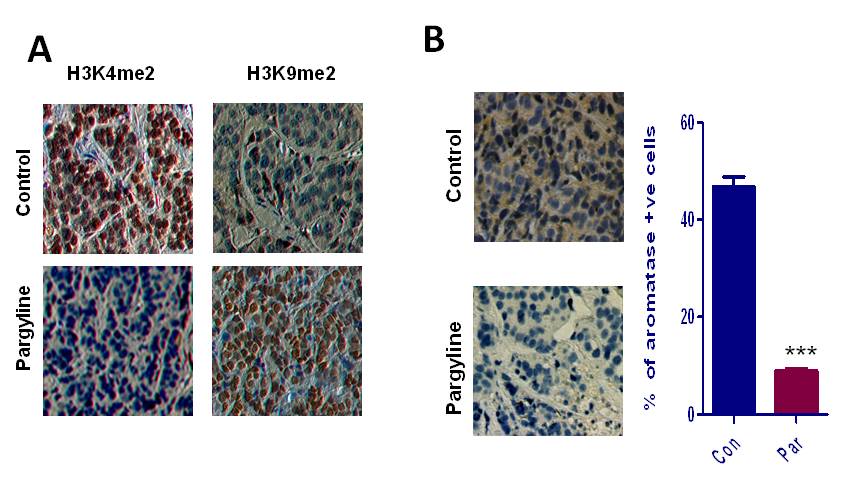

Supplement: Additional file 3 — Figure S3 showing that pargyline reduces oncogene-driven tumor growth in a postmenopausal xenograft model. (A) IHC analysis of indicated epigenetic marks was done on xenograft tumors that were treated with or without pargyline. (B) IHC analysis of aromatase expression was done on xenograft tumors that were treated with or without pargyline. ***P < 0.0001. [file bcr3229-S3.JPEG]

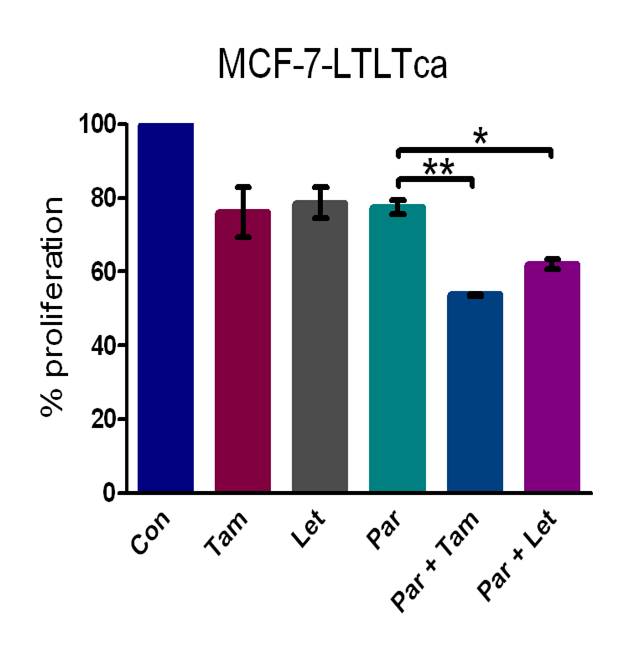

Supplement: Additional file 4 — Figure S4 showing that pargyline sensitizes therapy-resistant cells to hormonal therapy. Letrozole-resistant cells (MCF-7-LTLT) were treated with or without KDM1 inhibitor (pargyline) alone or in combination with letrozole (10-6 M) or tamoxifen (10-7 M) and cell proliferation was determined using the Cell Titer Glo assay. **P < 0.001, *P < 0.05. [file bcr3229-S4.JPEG]

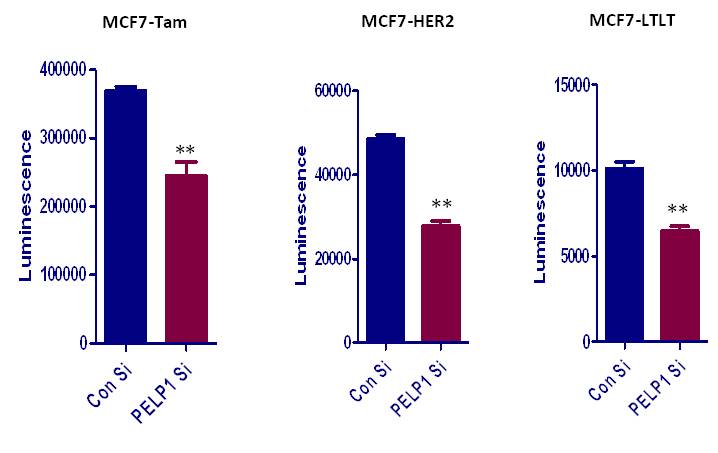

Supplement: Additional file 5 — Figure S5 showing that PELP1 knockdown reduces the growth of therapy-resistant cells. Therapy-resistant breast cancer cells were transfected with PELP1 siRNA (50 pmol) or control siRNA (50 pmol), and after 72 hours cell viability was measured by ATP assay (Cell Titer Glo ATP assay; Promega). **P < 0.001. [file bcr3229-S5.JPEG]

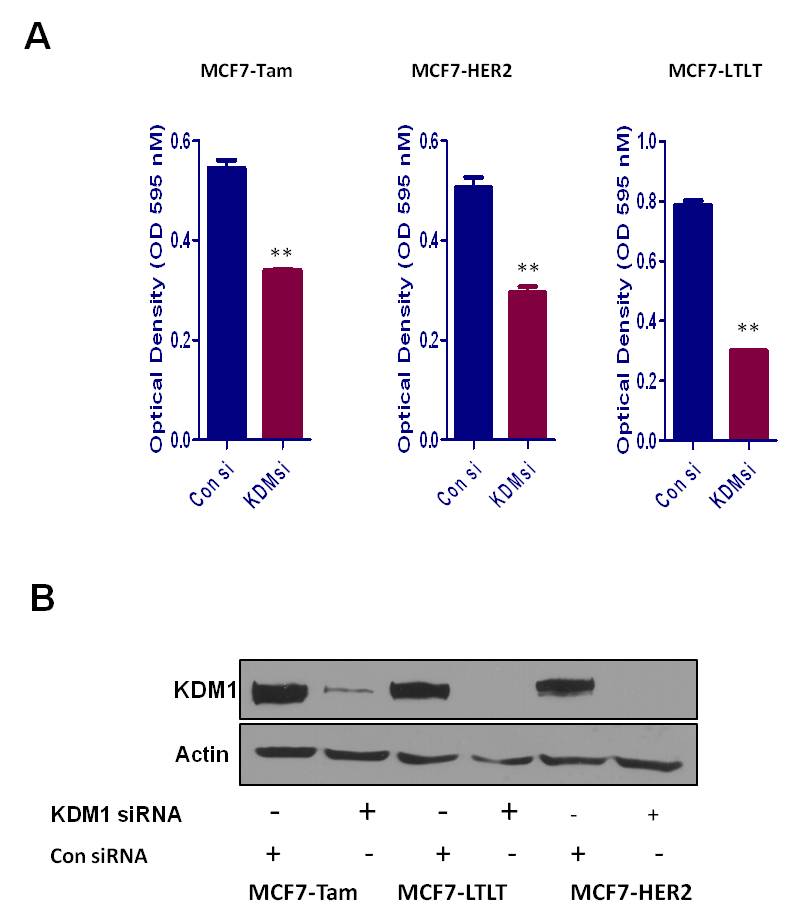

Supplement: Additional file 6 — Figure S6 showing that KDM1 knockdown reduces the growth of therapy-resistant cells. (A) Therapy-resistant breast cancer cells were transfected with KDM1 siRNA (50 pmol) or control siRNA (50 pmol), and after 24 hours were treated with tamoxifen (10-7 M). After 72 hours, cell viability was measured by MTT assay. **P < 0.001. (B) Western analysis of KDM1 using total lysates from control-siRNA and KDM1-siRNA transfected cells demonstrating the efficiency of KDM1 knockdown. [file bcr3229-S6.JPEG]
